# Análise da Sensibilidade e Especificidade dos Pontos de Corte para Frequência Cardíaca de Repouso em 6.794 Adolescentes Brasileiros: Um Estudo Transversal

**DOI:** 10.36660/abc.20210512

**Published:** 2021-07-15

**Authors:** Luana Anaisse Azoubel, Erika Ribeiro Carneiro, Nilviane Pires, Allan Kardec Barros, Carlos José Dias

**Affiliations:** 1 Universidade Federal do MaranhãoCentro de Prevenção de Doenças RenaisHospital Universitário do MaranhãoSão LuísMABrasilUniversidade Federal do Maranhão, Centro de Prevenção de Doenças Renais do Hospital Universitário do Maranhão, São Luís, MA - Brasil; 2 Universidade Federal do MaranhãoLaboratório de Adaptações Cardiorrenais Ao Exercício FísicoPinheiroMABrasilUniversidade Federal do Maranhão, Laboratório de Adaptações Cardiorrenais Ao Exercício Físico (LACE), Pinheiro, MA - Brasil; 3 Universidade Federal do MaranhãoDepartamento de Engenharia ElétricaLaboratório de Processamento de Informação BiologiaSão LuísMABrasilUniversidade Federal do Maranhão, Departamento de Engenharia Elétrica, Laboratório de Processamento de Informação Biologia, São Luís, MA - Brasil

**Keywords:** Frequência Cardíaca/fisiologia, Adolescente, Obesidade, Adiposidade, Pressão Arterial, Epidemiologia, Estudos Transversais/métodos

A frequência cardíaca de repouso (FCR) é uma medida acessível que pode refletir o equilíbrio entre o sistema nervoso simpático e o parassimpático.^1^ Níveis elevados de FCR estão associados a eventos cardiovasculares em adultos^2^ e fatores de risco cardiovascular, como sobrepeso,^3^obesidade abdominal e hipertensão arterial, foram associados a FCR.^4^ Portanto, obter um ponto de corte para FCR em crianças e adolescentes é essencial para um melhor rastreamento e subsequente seguimento clínico.

Além disso, a identificação precoce e o rastreamento das alterações cardiovasculares e da modulação autonômica cardíaca podem ser refletidas por medidas simples como a FCR, uma vez que adolescentes sedentários com obesidade abdominal apresentam menor variabilidade da frequência cardíaca (VFC) e consequentemente maior estimulação simpática, mantendo a FCR elevada e aumentando o risco de desenvolver lesões cardíacas ao longo da vida.^5^

Um método de verificação da modulação autonômica é através da VFC, que quando diminuída indica desequilíbrio autonômico e tem sido associada à mortalidade e a vários indicadores de risco cardiovascular (obesidade geral e abdominal, hipertensão e estilo de vida sedentário) em adolescentes.^6-8^ Um estudo anterior de Farah et al.,^9^ realizado com adolescentes do sexo masculino, demonstrou que os pontos de corte para VFC têm sensibilidade moderada a alta para detectar fatores de risco cardiovascular.^9^ Isso reforça a relevância de estudos que se propõem a estabelecer pontos de corte para variáveis de sinais biológicos, como a FCR e suas diferentes análises clínicas, com o objetivo de diagnosticar possíveis alterações cardiovasculares em crianças e adolescentes.

É isso que Farah et al.,^10^ propuseram neste estudo, ao estabelecer pontos de corte para FCR em adolescentes brasileiros e analisando associações entre os pontos de corte e fatores de risco cardiovascular. Foram avaliados 6.794 adolescentes (com idade de 10 a 19 anos), de ambos os sexos, e aferidas a pressão arterial e a FCR utilizando um aparelho oscilométrico. O índice de massa corporal (IMC) e a circunferência da cintura também foram avaliados. A curva ROC foi utilizada para analisar a sensibilidade e a especificidade, e as associações de FCR alta com fatores de risco cardiovascular foram analisadas através de regressão logística binária.^10^

Os principais achados desse estudo, além de determinar os pontos de corte da FCR, foram estabelecer a associação desses pontos de corte com fatores de risco cardiovascular, como obesidade abdominal, sobrepeso e hipertensão em meninos e meninas.

Um estudo de Christofaro et al.,^11^ encontrou uma associação entre frequência cardíaca e pressão arterial sistólica e diastólica em adolescentes, e apontou que para os meninos houve influência da obesidade abdominal e do IMC naqueles com frequência cardíaca mais elevada, mas isso não ocorreu nas meninas; assim, os autores sugeriram que padrões de distribuição de gordura corporal e variações hormonais podem influenciar esses achados.^11^ De fato, o padrão androide de distribuição de gordura corporal está mais associado a fatores de risco cardiovascular e os hormônios femininos (estrógenos) fornecem alguma proteção cardiovascular nas meninas.^12^

Além disso, como esperado, há um declínio na FCR com o aumento da idade, provavelmente devido à melhora da sensibilidade barorreflexa e função neural com a maturação sexual. De fato, ocorre um aumento progressivo da atividade cardíaca vagotônica em relação à atividade simpática durante a maturação, resultando em menor FCR no final da adolescência.^13^

Esse estudo tem dois outros destaques importantes: o número de participantes foi considerável (importante para fins epidemiológicos) e foi realizado em três regiões diferentes do país (Nordeste, Sudeste e Sul) possivelmente com etnias diferentes, embora isso não tenha sido relatado, sendo portanto representativo sob a perspectiva da variabilidade genética. Apesar desses resultados, o estudo apresenta limitações: não foi possível estabelecer um ponto de corte para as meninas de 10 a 14 anos e o motivo não foi explicado. Fatores externos que influenciam a frequência cardíaca (tabagismo, nível de atividade física, consumo de bebidas alcoólicas e cafeína) não puderam ser identificados, e é importante destacar essas limitações para estudos futuros.^14^

Para fins de triagem epidemiológica, o estabelecimento de pontos de corte que possibilitem a identificação do aumento do risco cardiovascular é de extrema importância, pois podem melhorar as estratégias de saúde pública para a população que presumivelmente apresenta maior risco. Além disso, no que diz respeito à população jovem, essas estratégias podem ser implementadas até mesmo nas escolas (incentivo à prática de esportes e alimentação saudável) com o reforço de um substrato fisiológico, a FCR, para ratificar a importância dessas estratégias. Portanto, por ser uma variável simples e ter baixo custo de mensuração, seu uso pode melhorar e aumentar a utilidade clínica para os profissionais de saúde na determinação do diagnóstico e prognóstico dos jovens brasileiros.
