# Supplementary material for: Análise da Sensibilidade e Especificidade dos Pontos de Corte para Frequência Cardíaca de Repouso em 6.794 Adolescentes Brasileiros: Um Estudo Transversal
Source: Arq Bras Cardiol. 2021 Jul 15;117(1):82–3. [Article in Portuguese] doi: 10.36660/abc.20210512 (PMC8294744; doi:10.36660/abc.20210512)
Supplement: Translation

# Translation: Analysis of Sensitivity and Specificity of Cutoff Points for Resting Heart Rate in 6,794 Brazilian Adolescents: A Cross-Sectional Study

**Authors:** Luana Anaisse Azoubel, Erika Ribeiro Carneiro, Nilviane Pires, Allan Kardec Barros, Carlos José Dias

**Affiliations:** 1 BrasilUniversidade Federal do Maranhão, Centro de Prevenção de Doenças Renais do Hospital Universitário do Maranhão, São Luís, MA - Brasil; 2 BrasilUniversidade Federal do Maranhão, Laboratório de Adaptações Cardiorrenais Ao Exercício Físico (LACE), Pinheiro, MA - Brasil; 3 BrasilUniversidade Federal do Maranhão, Departamento de Engenharia Elétrica, Laboratório de Processamento de Informação Biologia, São Luís, MA - Brasil

**Keywords:** Heart Rate/physiology, Adolescent, Obesity, Adiposity, Blood Pressure, Epidemiology, Cross-sectional studies/methods

Resting heart rate (RHR) is an accessible measure that may reflect the balance between the sympathetic and parasympathetic nervous system.^1^ Elevated RHR levels are associated with cardiovascular events in adults^2^ and cardiovascular risk factors, such as overweight,^3^ abdominal obesity and high blood pressure have been associated with high RHR.^4^ Therefore, obtaining a cutoff point for the RHR in children and adolescents is essential for better screening and subsequent clinical follow-up.

Furthermore, the early identification and screening of cardiovascular changes and cardiac autonomic modulation can be reflected by simple measures such as the RHR, since sedentary adolescents with abdominal obesity have lower heart rate variability (HRV) and, consequently, greater sympathetic stimulation, maintaining the RHR high and increasing the risk of developing heart damage throughout life.^5^

One method of verifying autonomic modulation is through the HRV, which when decreased indicates autonomic imbalance and has been associated with mortality and various cardiovascular risk indicators (general and abdominal obesity, high blood pressure and sedentary lifestyle) in adolescents.^6-8^ A previous study by Farah et al.,^9^ carried out with male adolescents, demonstrated that cutoff points for HRV have moderate-to-high sensitivity for detecting cardiovascular risk factors.^9^ This reinforces the relevance of studies that propose establishing cutoff points for biological signal variables, such as the RHR and its different clinical analyses, aiming to diagnose possible cardiovascular alterations in children and adolescents.

This is what Farah et al.^10^ proposed in this study, by establishing cutoff points for RHR in Brazilian adolescents and analyzing associations between cutoff points and cardiovascular risk factors. A total of 6,794 adolescents (aged 10 to 19 years), of both genders, were evaluated and blood pressure and RHR were measured using an oscillometric device. Body mass index (BMI) and waist circumference were also evaluated. The ROC curve was used to analyze sensitivity and specificity, and associations of high RHR with cardiovascular risk factors were analyzed by binary logistic regression.^10^

The main findings of this study, in addition to determining the RHR cutoff points, was to establish the association of these cutoff points with cardiovascular risk factors, such as abdominal obesity, overweight and high blood pressure in boys and girls.

A study by Christofaro et al.^11^ found an association between heart rate and systolic and diastolic blood pressure in adolescents, and it was pointed out that, for boys, there was an influence of abdominal obesity and BMI in those with a higher heart rate, but this did not occur in girls; thus, the authors suggested that patterns of body fat distribution and hormonal variations may influence these findings.^11^ In fact, the android body fat distribution pattern is more associated with cardiovascular risk factors and female hormones (estrogens) provide some cardiovascular protection in girls.^12^

Furthermore, as expected, there is a decline in RHR with increasing age, probably due to the improvement in the baroreflex sensitivity and neural function with sexual maturation. Indeed, there is a progressive increase in vagotonic cardiac activity in relation to sympathetic activity during maturation, resulting in lower RHR in late adolescence.^13^

This study also has two other important highlights: the number of participants was considerable (important for epidemiological purposes) and it was carried out in three different regions of the country (northeast, southeast and south), possibly with different ethnicities, although it was not reported, therefore being representative from the perspective of genetic variability. Despite these results, the study has limitations: it was not possible to establish a cutoff point for girls aged 10-14 years and the reason was not explained. External factors that influence heart rate (smoking, level of physical activity, consumption of alcoholic beverages and caffeine) could not be identified, and it is important to highlight these limitations for future studies.^14^

For epidemiological screening purposes, establishing cutoff points that allow the identification of increased cardiovascular risk is of utmost importance, since it can improve public health strategies for a population that is presumably at higher risk. Also, regarding the young population, these strategies can be implemented even in schools (encouraging the practice of sports and healthy eating) with the reinforcement of a physiological substrate, the RHR, to ratify the importance of these strategies. Therefore, since it is a simple and low-cost variable to measure, its use can improve and increase the clinical practicality for health professionals in determining diagnosis and prognosis of young Brazilians.
